# TIME to reduce agitation in persons with dementia in nursing homes. A process evaluation of a complex intervention

**DOI:** 10.1186/s12913-019-4168-0

**Published:** 2019-05-31

**Authors:** Bjørn Lichtwarck, Janne Myhre, Geir Selbaek, Øyvind Kirkevold, Anne Marie Mork Rokstad, Jūratė Šaltytė Benth, Sverre Bergh

**Affiliations:** 1 The Research Centre for Age-related Functional Decline and Disease, Innlandet Hospital Trust, Ottestad, Norway; 2 Institute of Health and Society, Faculty of Medicine, University of Oslo, Oslo, Norway; 3 Department of Public Health and Nursing, Faculty of Medicine and Health Sciences NTNU, Norwegian University of Science and Technology, Trondheim, Norway; 4 Norwegian National Advisory Unit on Ageing and Health, Vestfold Hospital Trust, Vestfold, Norway; 5 Departement of Health, Care and Nursing, Faculty of medicine NTNU, Norwegian University of Science and Technology, Gjøvik, Norway; 6 Faculty of Health Sciences and Social Care, Molde University College, Molde, Norway; 7 Institute of Clinical Medicine, Campus Ahus, University of Oslo, Oslo, Norway; 8 HØKH, Research Centre, Akershus University Hospital, Lørenskog, Norway

**Keywords:** Process evaluation, RE-AIM, Implementation, Case conferences, Dementia, Neuropsychiatric symptoms, BPSD, Non-pharmacological interventions, Complex interventions

## Abstract

**Background:**

The Targeted Intervention Interdisciplinary Model for Evaluation and Treatment of Neuropsychiatric Symptoms (TIME) has recently in a three-month cluster randomised controlled trial demonstrated reduction in agitation in nursing home residents with dementia. To ease replication and future implementation, and to clarify possible causal mechanisms, we performed a process evaluation of the intervention based on the RE-AIM framework (Reach, Effectiveness, Adoption, Implementation, Maintenance).

**Methods:**

An exploratory and a quasi-experimental design with mixed methods were used. The RE-AIM dimensions were explored by questionnaires to 807 staff members and 46 leading ward nurses in both the intervention nursing homes (INH) and the control nursing homes (CNH), before the start of the trial (baseline), and six and 12 months later. These questionnaires assessed data regarding the reach, effectiveness (staff level) and adoption dimensions. To assess implementation, we used a checklist for performance of the main components in TIME and analysed the minutes from 84 case conferences in the INH. To explore adoption and maintenance, five focus group interviews with 32 participants from the staff in the INH were conducted three to 6 months after the end of the trial.

**Results:**

Reach: On average 61% (SD 22) of the staff in each ward in the INH attended the training sessions. Effectiveness at staff level: There were no between-group differences throughout the study period for attitudes towards dementia, perceived competence or perception of mastery and social interaction. Adoption: 16 of the 17 INH completed the intervention. Implementation: 75% or more of the components of TIME were performed for 91% of the included residents. Maintenance: Most of the nursing homes used TIME three to 6 months after the end of the trial. An easy to grasp model and an engaged leadership facilitated the intervention and maintenance.

**Conclusions:**

A high degree of reach, adoption, implementation and maintenance contributed to the effectiveness of TIME at resident level. One other causal assumption of the effectiveness of TIME is the development in the staff of a new, shared and situated knowledge about each individual resident, not reflected by measurements in general knowledge and attitudes.

**Trial registration:**

The trial was registered January 6, 2016 with ClinicalTrials.gov (NCT02655003).

**Electronic supplementary material:**

The online version of this article (10.1186/s12913-019-4168-0) contains supplementary material, which is available to authorized users.

## Background

Nearly all persons living with dementia in nursing homes will develop neuropsychiatric symptoms (NPS) during the course of the condition [1, 2]. Of these symptoms, agitation including verbal and physical aggression and excessive motor activity, is one of the most frequent and persistent symptoms [1, 3, 4]. Agitation results in distress for the patients and their caregivers and is associated with reduced quality of life, referral to specialist health care and hospitalization, and a more rapid progression toward severe dementia and death [5–7]. The effect of psychotropic drugs used for agitation is modest, and their use is associated with major side-effects [8]. Treatment guidelines recommend non-pharmacological interventions for agitation as a first-line approach, even though the evidence of their effectiveness is contradictory [9–11]. Though some training interventions for nursing home staff aiming to reduce agitation in dementia have shown promising results [12, 13], most of these interventions have failed to demonstrate effectiveness at residential level [10, 14]. Nursing homes represent complex social systems which differ in their characteristics, sometimes changing during a trial. The settings will affect both the content of an intervention (i.e. standardisation) and the implementation process [15, 16]. These characteristics of the care settings pose important challenges for both the intervention and the implementation process to be efficacious [16, 17]. Findings from a systematic review to identify features of effective dementia educational programs by Surr et al. (2017), emphasised that for learning to take place, theory must underpin practice-based learning, involve active face-to face participation and provide a structured tool to guide practice [17]. To address the challenges in complex systems and improve the adoption of interventions, Hawe et al. (2004) recommended allowing for a certain degree of flexibility and the adaption of the intervention to the local context (i.e. a more pragmatic design in trials) [16].

In a recent three-month cluster-randomized controlled trial conducted in Norway, The Targeted Interdisciplinary Model for Evaluation and Treatment of Neuropsychiatric Symptoms (TIME), was compared to usual care supplemented by a brief educational intervention [18]. In this trial clinically significant reduction in agitation (primary outcome) and in symptoms of delusions, depression, and disinhibition, as well as improved quality of life (secondary outcomes) was found in favour of the intervention group [19]. An important question regarding evidence-based interventions like TIME, is how to obtain a simple, effective, and sustainable implementation [10, 20].

### Description and development of TIME

The published protocol paper for the trial and the manual at the TIME website (English version) give a complete description of TIME, the model’s development, and its theoretical underpinnings [18, 21]. TIME is an evidence based, multicomponent, biopsychosocial intervention to be used by the nursing home staff and physicians. Principles from cognitive behavioural therapy (CBT) and person-centred care form the theoretical base for TIME [22, 23]. TIME is divided in three overlapping phases: a registration and assessment phase (Table 1); a guided reflection phase, including case conferences (Table 2); and an action and evaluation phase. The actions to take in this last phase are to be described as SMART. SMART stands for Specific, Measurable, Actual, Realistic and Time framed [30]. The evaluation of these actions is performed using the same clinical scales and observation forms as described in the first phase. Because the actions and treatment measures are supposed to be tailored to each resident, they will display great variations between patients. In this way, TIME serves as a guide for the staff to create actions and treatment measures that are customised to the resident. These three phases are in line with reviews describing the “state of the art” for management of NPS [9, 31]. A qualitative study exploring the staffs’ experiences with TIME showed that the model shifted the way of learning for the staff from a traditional to a shared, innovative and reflection-based learning at work using the case conferences. The staff experienced increased coping when approaching complex problems [32].

**Table 1:** Registration and assessment phase

Checklist for the registration and assessment phase of TIME			
Activity	Target symptoms: Agree on the primary challenges for the patient using the Neuropsychiatric Inventory-Nursing Home Version (NPI-NH) to define precise target symptoms for the assessment		
	Observation of the target symptoms using a 24-h observation form	Staff	Responsible
	NPI-NH^a^ to assess other neuropsychiatric symptoms	Staff	
	CSDD^b^ or another scale to assess possible symptoms of depression	Staff	
	Physical assessment	Nursing home physcian	
	Review of medication	Nursing home physcian	
	MOBID-2^c^ or another assessment scale to assess possible pain	Staff Nursing home physcian	
	CDR^d^ and/or the MMSE^e^ to assess the dementia stage	Staff Nursing home physcian	
	PSMS^f^ or another assessment scale to assess activities in daily life	Staff	
	Collection of resident life history, including preferences and resources, using an optional questionnaire	Staff interview the resident (if possible) and/or the next of kin	
	Make an appointment, i.e., set the date, time and place for the case conference	Staff/TIME administrator	

Notes: ^a^Neuropscychiatric Inventory Nursing Home version (NPI-NH) [63]; ^b^Cornell Scale of Depression in Dementia (CSDD) [24, 25], ^c^Mobilisation-Observation-Behavior-Intensity-Dementia Scale (MOBID-2) [26], ^d^Clinical dementia Rating Scale (CDR) [27]; ^e^Mini-Mental State Examination (MMSE) [28], ^f^Physical Self-Maintenance Scale (PSMS) [29]

**Table 2:** Agenda and timeframe for the case conference

Agenda for the case conference 60–90 min				
Activity	Preparation: Convene a meeting and prepare a meeting room with a blackboard or similar facilities (projector, if available). Check that a flip pad and markers are available	TIME administrators: One is the chairman for the meeting. One takes notes on the whiteboard. One writes the minutes on the 5-column sheet.		Responsible
	1. Status Report: Personal history and main points from the patient’s medical record are presented.	10–15 min	Decide in advance who should prepare and present the patient’s personal history and the main points from the medical record.	
	2. Create a problem list	10 min	Staff (as many as possible should attend the conference) The leading registered nurse and the nursing home physician should attend the conference, if possible.	
	3. Prioritise problems from the list			
	4. Draw a 5-column sheet on the whiteboard: facts – interpretations (thoughts) - emotions – actions – evaluation	60 min		
	5. Describe facts from the registration and assessment phase: one problem at a time			
	6. Suggest interpretations – guided discovery – discuss and reflect on them			
	7. Describe any emotions experienced by the staff – with interpretations by the staff			
	8. Suggest SMART^a^ actions – based on the interpretations – decide how and when to perform an evaluation of the actions			
	9. Summarise interpretations and actions – close the meeting	5–10 min	TIME-administrator (chairman)	

Notes: ^a^SMART (Specific, Measurable, Actual, Realistic, Time-framed) [30]

### Implementation strategies used in the trial

The published study protocol gives a detailed description of the control and intervention phases in the trial [18]. The implementation process started with an information meeting (1.5 h) with leaders (i.e. leading ward nurses, managers, and physicians) from the nursing home and the health and care authorities in the municipality to introduce TIME and the necessary steps for its implementation. Before randomisation, three nurses from each ward in both the intervention nursing homes (INH) and the control nursing homes (CNH) were offered a three-hour lecture about the trial and the assessments instruments to be used for the residents. After randomisation, a two-hour lecture covering dementia and NPS was given to the staff in both the INH and the CNH. For the staff in the CNH, this lecture represented the education-only intervention. For the remainder of the trial, the staff of the CNH continued with practice as usual. An additional training program with a three-hour lecture and role play about TIME was offered to the staff in the INH. The responsibility for the implementation of TIME was given to three nurses in each ward in the INH together with the leading ward nurse. These three nurses were given the title TIME administrators. The leading ward nurse selected the TIME-administrators for each ward, based on their interest in professional development, their legitimacy with the rest of the staff, and not having a part-time job. The TIME-administrators received three additional hours of training. A specialist nurse from the education and training team attended the TIME administrators’ first case conference on their first resident in their nursing home in a supervisory capacity. The education and training team in the TIME trial consisted of five specialist registered nurses in geriatrics or old age psychiatry and one physician with special competence in nursing home medicine. All members of the team were familiar with TIME and had used the model for some years in real-world clinical settings. In addition to the training sessions, all the staff members in the INH were given the TIME manual and they had access to a website with assessment sheets, an educational film and other educational material to support the intervention [21, 33].

The intervention with TIME can be regarded as a pragmatic trial due to relevant clinical inclusion criteria and the primary outcome measure, the low involvement of the researchers in the implementation process, the low use of other external resources, the rather modest time allocated for educational purposes and the moderate degree of flexibility for adaption of the intervention to the context of the nursing homes [34].

### The need for process evaluations of complex interventions

TIME can be regarded as a complex intervention because it is composed of multiple components with the potential for interactions between them and with a number of possible outcomes [35]. Few studies of complex interventions in nursing homes report from the implementation process and to what degree the interventions were adopted and sustained over time [36–40]. A common conclusion in these reports is that management support and organisational factors are the main issues in the implementation process. The comprehensive review of the effect of staff training on staff outcomes by Spector et al. in 2016 concluded that many staff factors are difficult to change [40]. One important question for health leaders and policy makers is to what extent the intervention is flexible, easy to implement and can be adapted to the local context. Furthermore, a process evaluation is important to guide projects which aim to replicate the trial. And finally, it is necessary to clarify possible causal mechanisms to better inform and prepare further dissemination and implementation of the intervention on a larger scale [20, 41].

### The RE-AIM framework for evaluation of complex interventions

The Medical Research Council (MRC) defines an overarching framework for the development and evaluation of complex interventions emphasizing the value of combining both quantitative and qualitative data in the process [42]. In the MRC guidance the RE-AIM framework is described as a useful framework for assessing the overall implementation impact of interventions. RE-AIM is an acronym for five evaluation dimensions: Reach (the number, proportion and characteristics of the target population that participated), Effectiveness (the ability to change desired outcomes), Adoption (the number or proportion of wards who adopted the intervention), Implementation (how well the intervention was delivered as designed), and Maintenance (how well program effects are maintained and the continued use of the program) [43–45]. In this report, we have followed the recommendations from the MRC guidance and performed a process evaluation of the intervention with TIME using the RE-AIM evaluation framework.

### Aims

The primary aim of the study was to perform a process evaluation of the intervention of TIME to ease replication and future implementation. As a part of this evaluation, we wanted to explore what factors inhibited or promoted the implementation. Secondly, we aimed to clarify possible causal mechanisms for the effectiveness of TIME at resident level.

## Methods

### Design

We used a quasi-experimental (a pre-test post-test, control group) and a qualitative exploratory design. Mixed methods of the convergent parallel type according to Creswell and Clark were used [46]. In this type of mixed-method study, qualitative data and quantitative data are collected in the same study and with equal priority. The data are analysed separately but integrated in the overall interpretation of the results.

### Sites and participants

A total of 89% of nursing homes in Norway are run by the municipalities and 11% are run by non-profit organisations or private enterprises. According to national specific regulations, each nursing home must have an administrative manager, a registered nurse responsible for the nursing care, adequate staffing to ensure necessary care for the residents, and a nursing home physician [47]. Most physicians working in nursing homes are general practitioners in part-time positions. Sixty-three municipalities with a total of 130 nursing homes located in the north, middle, and south-eastern parts of Norway were invited to take part in the cluster-randomised controlled trial (TIME). The choice of this convenience sample was made because of resource reasons to minimise travel time and costs for the research and educational group and, at the same time, strive for a representative sample of NH. The approach was effectuated by an e-mail invitation to the manager of the elderly care department of each municipality. In the e-mail intervention, we clarified that nursing homes already using TIME, nursing homes engaged in other research projects and nursing homes that primarily offered short-term care could not participate in the trial. The research team arranged information meetings for managers and physicians from nursing homes in 32 municipalities who responded positively to the invitation. In these municipalities there were 63 nursing homes. Participation in the study for the municipalities and their NH, as is the rule for most other RCTs, was based on interest and was voluntary [48]. After the information meetings 12 of the 32 municipalities declined to participate in the trial. The reasons they gave for their refusal were, ‘being engaged in other research projects’ (five), ‘lack of resources and being engaged in internal educational projects ‘(five) and ‘already using TIME’ (one). One municipality did not give any reason for the refusal to participate. Finally, a total of 33 nursing homes from 20 municipalities accepted our invitation to take part in the trial. After baseline assessments, 17 nursing homes with 104 included residents from 22 wards were randomly assigned to the intervention group (INH), and 16 nursing homes with 125 included residents from 24 wards were randomly assigned to the control group (CNH) [49].

In this process evaluation of the trial, 797 staff members and 22 leading ward nurses from the INH and 889 staff members and 24 leading ward nurses from the CNH were invited to participate in the survey by receiving questionnaires at baseline. The questionnaires were distributed by e-mail, using a work address list of all employees including both regular and temporary staff members, in each ward who had residents included in the trial. The five focus groups consisted of 32 staff members, leaders, and physicians from 11 of the 17 INH. The nursing homes that were represented in the groups were selected randomly from the pool of INH to minimize selection bias [50]. We did not conduct focus groups in all the 17 INH to minimise travel time and costs for the research group. To achieve information-rich cases and a purposeful sample, the leading ward registered nurse in each nursing home selected who of the staff would attend the focus groups [51]. The criteria for the selection of participants were that they should be familiar with TIME and be able to promote views in a group discussion. A detailed description of the composition of the focus groups can be found in the Additional file 2 (Methods used for the collection of qualitative data and their analyses).

### Data collection

#### Questionnaires

The dimensions from the RE-AIM framework were assessed using questionnaires to the staff and leaders. An overview of the questionnaires, their timeframe, the target population (i.e. staff members or leadership in INH or CNH) and their relation to the RE-AIM dimensions is presented in Table 3. The complete set of questionnaires is presented in the Additional file 1 (Questionnaires).

**Table 3:** Overview of questionnaires distributed to the staff and the leading ward registered nurses, timeframe, and their relation to the RE-AIM-framework^a^

What is assessed?	Questionnaire	Corresponding dimension of the RE-AIM framework	Time frame	Respondents in the nursing homes (NH)
Proportion of staff members participating in education and training sessions	A registration form	Reach: proportion of staff in INH that participated in the intervention during the trial	At the start of the intervention during education sessions	All staff members in intervention nursing homes (INH)
Attitudes towards persons with dementia, mastery, social interaction, job satisfaction and self-assessment of competence with neuropsychiatric symptoms (NPS)	ADQ^b^, QPS-Nordic^c^ and The Competence Questionnaire (a self-developed questionnaire for assessment of competence with NPS)	Efficacy: outcomes regarding knowledge, skills and/or attitudes of the staff in NH	1 month before (baseline), and 6 and 12 months after the start of the intervention	All staff members in control nursing homes (CNH) and in INH
Clinical routines in place in NH, i.e., questions assessing daily routines of practice for assessment and treatment of NPS at ward level	The Current Practice Questionnaire (a self-developed questionnaire based on evidence-informed best practice for assessment and treatment of NPS)	Adoption: proportion of wards that will adopt the intervention Maintenance: extent to which the model is sustained over time	1 month before (baseline) and 6 and 12 months after the start of the intervention	Leading ward registered nurse in INH and CNH
Fidelity to the main components in the model	The Fidelity Questionnaire: (Interview of TIME administrators by telephone using a checklist based on the components in the TIME manual)	Implementation: extent to which the intervention is implemented	3 brief interviews, the first one 3 weeks after the start of the intervention and then at 1-month intervals	TIME administrators in INH
Organizational structure in the nursing homes: size of wards, type of unit, number of staff, etc.	A registration form	Implementation: possibility to assess and analyse implementation barriers and facilitators	At the start of the intervention	Leading ward registered nurse in INH and CNH

^a^RE-AIM Reach Effectiveness Adoption Implementation Maintenance [43]; ^b^ADQ The Approach to the Dementia Questionnaire [52]; ^c^QPS-Nordic The General Nordic Questionnaire for Psychological and Social Factors at Work [53]

Using a questionnaire, we asked the staff to provide their demographic information, such as age, employment relationship, percentage of full time engagement, number of years of experience in health-related jobs, and number of years of health-related continuing education. Organizational and structural factors of the NH wards were obtained from the leading ward nurses: type of ward (special care ward or regular ward), the ward size (number of residents and the number of full-time employment equivalent), the number of staff present per resident during daytime, and the number of hours the nursing home physician is available per resident per week.

The following three questionnaires were administered to the staff before the start of the intervention (i.e. before randomisation of the nursing homes), and six and 12 months later, in both INH and CNH (name or acronym of questionnaires, RE-AIM dimensions): the Approaches to Dementia Questionnaire (ADQ, Effectiveness) [52, 54], the General Nordic Questionnaire for Psychological and Social Factors at Work regarding mastery and social interaction (QPS-Nordic, Effectiveness) [53], and a brief self-developed questionnaire assessing perceived competence for individual staff members regarding NPS (Competence Questionnaire, Effectiveness). Only those of the staff who answered a questionnaire at baseline received a follow-up questionnaire at 6 months, and only those who answered at 6 months received a follow-up questionnaire at twelve months.

The QPS-Nordic has been validated in a Norwegian version and has acceptable construct and content validity as well as internal consistency and test-retest reliability [53, 55]. The QPS-Nordic consists of 13 subscales covering essential social and psychological factors at work. The two subscales labelled perception of mastery and perception of social interaction were used. To respond, staff members chose a value on a five-point Likert scale ranging from 1 (very seldom or never) to 5 (very often or always) to indicate how appropriate each statement was for them. Each subscale score for six items ranged from 6 to 30.

The ADQ assesses general attitudes to dementia. The questionnaire has acceptable content and construct validity as well as internal consistency, inter-rater and test-retest reliability [54]. ADQ has been translated to a Norwegian version [52]. The questionnaire consists of 19 statements, where respondents indicate on a five-point Likert scale from 1 (strongly agree) to 5 (strongly disagree) the extent to which each statement was in accordance with their attitudes. The total sum score ranges from 19 to 95, with higher scores signifying more positive attitudes. A factor analysis resulted in two domains: the “hope” attitude (8 items, range 8–40) and the “person-centred” attitude (11 items, range 11–55) [54].

The Competence Questionnaire consisted of five statements. The respondent indicated how appropriate each statement was for their situation on a seven-point Likert scale from 1 (very low competence) to 7 (very high competence) giving a total sum score between 7 and 35. Competence was defined in the questionnaire as a composition of the concepts “knowledge” and “skills” [56].

A questionnaire assessing the performance of the main 10 components of the TIME (Fidelity Questionnaire, Implementation) were addressed by a brief telephone interview to one of the TIME administrators in the INH for each included resident, up to three times during the trial with 3 to 4 weeks interval. Each component was given a score of 1 if it was performed, except for the performance of the case conference which was given a score of 8, and the evaluation procedure which was given a score of 4. The weighting of the score was based on the presumed time to perform each component. The sum score of the questionnaire thus had a score ranging from 0 to 20, where a higher score indicates greater fidelity to the model. A score of 20 for a resident, means that 100% of the components for the resident was performed.

A self-developed questionnaire for assessment and treatment routines of NPS at ward level (Current Practice Questionnaire, Adoption and Maintenance) were distributed to the leading ward nurses before the start of the intervention, and six and 12 months later, in both the INH and CNH. This questionnaire was developed by the research team based on the TIME manual and on evidence-informed best practice [9, 21, 31]. Respondents indicated how appropriate each statement was for their situation on a five-point Likert scale from 1 (very seldom or never) to 5 (very often or always). The questionnaire consists of 13 items, giving a sum score from 13 to 65 where a higher score indicates a better practice for assessment and treatment of NPS.

A registration form to assess participation of staff, leaders and physicians in education and training sessions were used (Reach). The Reach fraction for the attendance to the training sessions was calculated by the number of participants per ward who attended the training sessions divided by the number of potential participants (i.e. the total number of regular and temporary staff in the ward) [44].

### Focus groups and minutes from case conferences

To explore adoption and maintenance, five focus groups interviews with 32 of the caregivers, leaders, and physicians from 11 of the 17 INH were conducted 3 to 6 months after the end of the 3 month intervention [57]. To further assess implementation, we collected the minutes from 84 of the 85 performed case conferences in the INH. A detailed description of the selection and the composition of the focus groups and the collection of the minutes can be found in the Additional file 2 (Methods used for the collection of qualitative data and their analyses).

### Data analysis

#### Statistical analysis

Data were presented as means and standard deviations (SD) or frequencies and percentages, as appropriate. Differences between the two groups in the sum scores for items from the questionnaires were assessed by a linear mixed model with random effects for wards, controlling for possible intra-ward correlations. Fixed effects for time, group allocation and the interaction between these two were included into the model. A significant interaction implies differences between the groups throughout the study period. The results were presented as means and 95% confidence intervals estimated at each time point within the groups with *p*-values quantifying the between- and within-groups differences. Statistical analyses were performed using SPSS v24 and SAS v 9.4. The results with p-values below 0.05 were considered statistically significant.

#### Qualitative analysis

For the analysis of the interviews of the focus groups, thematic content analysis was used. Thematic content analysis is a method with the purpose to identify, analyse, and report patterns and themes in qualitative data [58, 59]. The aim is to provide a systematic description of both the manifest and latent content of the data, and in the end to evolve new concepts and understanding of phenomena [58]. Our approach to the coding process was mainly inductive and data-driven [58]. Preliminary analyses were conducted after each group, and when no obvious new meanings units or new sub-themes occurred in the last interview, we considered having met saturation of the data [60]. Qualitative analyses were performed using the word processor Word creating new documents based on coding of meaningful units, subthemes, and themes in the text to be analysed. Detailed description of this process can be found in the Additional file 2.

The documentary analysis of the minutes from the case conferences were performed by using structuring content analysis [61, 62]. This analysis looks for types or formal structures in the documents and uses preformed categories as codes to analyse frequencies and different degrees of quality in a category. The analysis is mainly deductive. Our categories were the main structuring components of the case conferences used in TIME, i.e. use of a problem list, selection of a prioritized problem, the degree of details in the description of the prioritized problem, the overall understanding and use of the five columns, actions described as SMART, and the degree of details in the description of the evaluation procedures of treatment actions. A detailed description of both the qualitative analysis methods, can be found in the Additional file 2.

## Results

Eight-hundred and seven (48%) of the staff, 366 from the INH (46%) and 441 (50%) from the CNH, accepted to participate in the survey by answering the baseline questionnaires. At follow-up 6 months from baseline, the response rates among those who answered at baseline were 188 (51%) and 222 (50%) in the INH and the CNH respectively. The response rate at 12 months follow-up, among those who answered at baseline, was 141 (39%) and 156 (35%) in INH and CNH, respectively.

On behalf of their wards, 21 (95%) of the leading ward nurses from INH and 24 (100%) from the CNH answered at baseline the Current Practice Questionnaire. At 6 months from baseline the response rate was 21 (95%) and 23 (96%) in INH and CNH, respectively. The response rate at 12 months was 20 (91%) and 22 (92%) in INH and CNH, respectively.

The Fidelity Questionnaire for the INH and the registration form to assess participation in education and training sessions was completed for 100% of the wards in the INH and the CNH.

Staff and ward characteristics are presented in Table 4. Staff and ward characteristics for those who participated in the focus groups are presented in Additional file 2**.**

**Table 4:** Baseline characteristics of the nursing staff (*N* = 804^a^) and the wards (*N* = 46). Values are numbers (%), unless otherwise specified

Characteristics	INH^b^	CNH^c^	Total
Age (years)			
≤29	65 (17.9)	87 (19.8)	152 (18.9)
30–49	147 (40.4)	176 (40.0))	323 (40.2)
≥50	152 (41.8)	177 (40.2)	329 (40.9)
Employment relationship			
Regular staff	335 (92)	397 (90.2)	732 (91)
Temporary staff	29 (8.0)	43 (9.8)	72 (9)
Percent of full time engagement			
<25	37 (10.2)	69 (15.7)	106 (13.2)
25–49	20 (5.5)	25 (5.7)	45 (5.6)
50–74	86 (23.6)	97 (22.0)	183 (22.8)
75–100	221 (60.7)	249 (56.6)	470 (58.5)
Working experience in years in health-related job			
0–1	10 (2.7)	13 (3.0)	23 (2.9)
1–5	57 (15.7)	83 (18.9)	140 (17.4)
6–10	67 (18.4)	90 (20.5)	157 (19.5)
11–15	68 (18.7)	78 (17.7)	146 (18.2)
>15	162 (44.5)	176 (40.0)	338 (42.0)
Health-related education			
3 years or more	91 (25)	121 (27.5)	212 (26.4)
Less than 3 years	223 (61.3)	247 (56.1)	470 (58.5)
No relevant health-related education	50 (13.7)	72 (16.4)	122 (15.2)
Relevant continuing education			
Yes	102 (28)	110 (25)	312 (26)
No	262 (72)	330 (75)	592 (74)
Wards			
Regular ward	3 (14)	9 (37)	12 (26)
Special care unit	19 (86)	15 (63)	34 (74)
Residents per ward. Mean (SD^d^)	22.5 (8.5)	22.8 (7.8)	22.7 (8.0)
Number of Full Time Equivalents per ward. Mean (SD), *n* = 45	25.1 (9.8)	22.1 (6.9)	23.5 (8.4)
Staff per ward per resident on day shift. Mean (SD), *n* = 45	0.36 (0.11)	0.33 (0.07)	0.35 (0.09)
Hours per resident per week for nursing home physician, Mean (SD), *n* = 45	0.34 (0.12)	0.32 (0.20)	0.33 (0.18)

Notes: ^a^ *N* = 804, 3 of the staff members did not answer the part of the questionnaire concerning age, education, employment etc. but answered some of the other questionnaires, with *N* = 807 for those questionnaires (see Table 5). ^b^INH. Intervention Nursing Homes; ^c^CNH. Control Nursing Homes ^d^SD; Standard Deviation

### Reach

The mean reach fraction for the attendance to the training sessions in the INH were 61% (SD 22) and 51% (SD 24) for the brief educational session in the CNH. In the INH, seven out of 17 nursing home physicians (41%) and 14 out 22 (64%) of the leading ward nurses attended the training sessions. Of the 78 TIME administrators, 43 (55%) were registered nurses or had an education equivalent to at least 3 years of college or university education in the field of elderly or health care and 35 (45%) were auxiliary nurses. On average there were 8 (SD 2.9) staff members participating in the case conferences ranging from 3 to 19. Nursing home physicians and leading ward nurses attended 27 (32%) and 42 (49%) of the case conferences, respectively.

### Effectiveness

The results from the questionnaires on the effectiveness at staff level are presented in Table 5. There were no statistically significant group differences throughout the study period for the sum scores for attitudes towards dementia, perceived competence or perception of mastery and social interaction for attitudes towards dementia, perceived competence or perception of mastery and social interaction. The sum score for attitudes towards dementia increased from baseline to twelve months in the INH (*p* = 0.029), and in the CNH (*p* = 0.006). The sub-score for hope attitudes increased from baseline to twelve months in the intervention group (*p* = 0.012) and from baseline to 6 months (*p* = 0.003) and to twelve months (*p* = 0.001) in the control group. The sum score for perceived competence for individual staff members regarding NPS increased from baseline to twelve months in the INH (*p* = 0.002). There was a reduction in the social interaction score for the CNH from baseline to 6 months (*p* = 0.009).

**Table 5:** Outcome measures on effectiveness at staff level and change in current practice at ward level (adoption). Mean scores and 95% confidence intervals (CI) before start of the intervention (Month 0), 6 and 12 months, and results of linear mixed models

Group	Within-group values								Difference between groups		
	Month 0		Month 6		Month 12		p-values for change		p-values		
	N	Mean (95% CI)	N	Mean (95% CI)		Mean (95% CI)	Month 0 to Month 6	Month 0 to Month 12	Month 0	Month 6	Month 12
*ADQ sum score*											
Intervention	364	71.4 (70.6; 72.1)	188	71.8 (70.8; 72.7)	141	72.7 (71.6; 73.8)	0.454	0.029	0.434	0.817	0.825
Control	441	71.0 (70.3; 71.7)	222	71.7 (70.8; 72.6)	156	72.5 (71.5; 73.5)	0.130	0.006			
*ADQ hope score*											
Intervention	364	26.1 (25.1; 26.8)	188	26.2 (25.5; 27.0)	141	27.1 (26.3; 27.8)	0.809	0.012	0.653	0.179	0.884
Control	441	25.9 (25.4; 26.5)	222	26.9 (26.2; 27.6)	156	27.2 (26.5; 28.0)	0.003	0.001			
*ADQ person centred score*											
Intervention	365	45.3 (44.8; 45.7)	188	45.6 (45.0; 46.2)	141	45.6 (44.9; 46.2)	0.346	0.377	0.499	0.053	0.460
Control	441	45.0 (44.6; 45.5)	222	44.8 (44.2; 45.3)	156	45.2 (44.6; 45.9)	0.358	0.572			
*The Competence Questionnaire Score*											
Intervention	365	28.5 (27.9; 28,6)	188	29.1 (28.5; 29.8)	141	29.5 (28.8; 30.3)	0.035	0.002	0.781	0.337	0.102
Control	441	28.3 (27.8; 28.9)	222	28.7 (28.1; 29.3)	156	28.7 (29.0; 29.4)	0.230	0.273			
*QPS-Nordic (mastery score)*											
Intervention	366	23.6 (23.5; 24.4)	188	24.0 (23.8; 24.6)	141	24.1 (23.5; 24.6)	0.776	0.700	0.456	0.812	0.850
Control	441	24.2 (23.8; 24.6)	222	23.9 (23.5; 24.4)	156	24.1 (23.6; 24.7)	0.300	0.874			
*QPS-Nordic (social interaction score)*											
Intervention	366	23.0 (22.3; 23.6)	188	23.0 (22.2; 23.7)	141	22.7 (21.9; 23.5)	0.991	0.441	0.478	0.473	0.547
Control	441	23.3 (22.7; 23.9)	222	22.6 (21.9; 23.3)	156	23.0 (22.3; 23.8)	0.009	0.423			
*The Current Practice Questionnaire*											
Intervention	21	47.0 (44.3; 49.7)	21	52.4 (46.7; 55.2)	20	51.8 (49.0; 54.6)	<0.001	0.001	0.912	0.338	0.337
Control	24	46.8 (44.3; 49.3)	23	50.6 (48.0; 53.2)	22	49.9 (47.2; 52.6)	0.006	0.026			

Notes: ADQ, Attitudes to Dementia Questionnaire, sum score, range 19–95; ADQ hope score, range 8–40; ADQ person centred score, range 11–55; The Competence Questionnaire, range 7–35; QPS-Nordic, General Nordic Questionnaire for Psychological and Social Factors at Work. mastery score, range 6–30: QPS-Nordic, social interaction score range 6–30; The Current Practice Questionnaire, range 13–65

### Adoption

Sixteen of the 17 INH completed the intervention. One INH, with one ward and seven residents included, withdrew from the trial approximately 2 weeks after the start of the intervention (i.e. after both the randomisation process and the training sessions). The reason given for this by the leading ward nurse, was an extremely high and prolonged level of sickness leave in the staff. It was not possible for her to assign someone from the staff to be interviewed for the assessments of the residents at eight and 12 weeks. Of the 22 wards in the INH that completed the study, all but two wards performed more than 75% of the components in TIME for their included residents as measured by the Fidelity Questionnaire. These two wards completed all or part of the first phase of TIME but performed no case conferences or evaluations of treatment actions. For both these wards the reasons given from the leaders for this reduced adoption of TIME, were lack of time and resources.

The INH and the CNH were not statistically different throughout the study period regarding adherence at ward level to recommended clinical practice for the assessment and treatment of NPS, as measured by the Current Practice Questionnaire (See Table 5). Both groups did however increase their scores from baseline to six and twelve weeks indicating better adherence to - and maintenance of - recommended clinical practice.

### Implementation

Overall, the staff performed 75% or more of the components in TIME for 91% of the included residents as measured by the Fidelity Questionnaire (i.e. a score ranging from 15 to 20). The INH performed a case conference in 91% of the included residents. The number of included residents with completed main components in TIME, is presented in Table 6. No nursing home performed more than one case conference for each resident during the 3 month trial. The average number of weeks that elapsed from intervention initiation (i.e. the randomisation date) to the performance of the case conferences was 5.0 (SD 1.8).

**Table 6:** The number of included residents with completed main components in TIME. Values are numbers (%)

Main components in TIME		Number (%) of included residents for whom the component was performed
1	Use of a 24 h observation form for symptoms or behaviour, *N* = 96^a^	95 (99)
2	Assessment of neuropsychiatric symptoms using the NPI-NH^d^, *N* = 96	90 (94)
3	Assessment of personal life story, *N* = 96	95 (99)
4	Assessment of depression using the CSDD^e^ or equivalent scale, *N* = 96	90 (94)
5	Assessment of activities in daily life using the PSMS^f^ or equivalent scale, *N* = 96	93 (96)
6	Pain assessment, *N* = 96	90 (94)
7	Assessment of degree of dementia with CDR^g^ or equivalent scale, *N* = 96	96 (100)
8	Examination by a physician, *N* = 96	88 (92)
9	Performance of a case conference, *N* = 93^b^	85 (91)
10	Systematic evaluation of treatment measures, *N* = 90^c^	60 (67)

Notes: ^a^N = 96, 104 residents were included in the trial in the intervention nursing homes, 7 residents were lost to follow up as 1 nursing home withdrew from the trial 2 weeks after baseline measures, and 1 resident died shortly after baseline; ^b^ *N* = 93, as for ^a^ *N* = 96 and in addition 2 residents died and 1 resident moved from the nursing home before the case conference was scheduled; ^c^ *N* = 90, as for ^b^ *N* = 93, and in addition 3 residents died before the evaluation was scheduled; ^d^NPI-NH, Neuropsychiatric Inventory-Nursing Home Version [63]; ^e^CSDD, Cornell Scale for Depression in Dementia [24]; ^f^PSMS, Physical Self-Maintenance Scale [29]; ^g^CDR, Clinical Dementia Rating Scale [27]

**Fig. 1:**
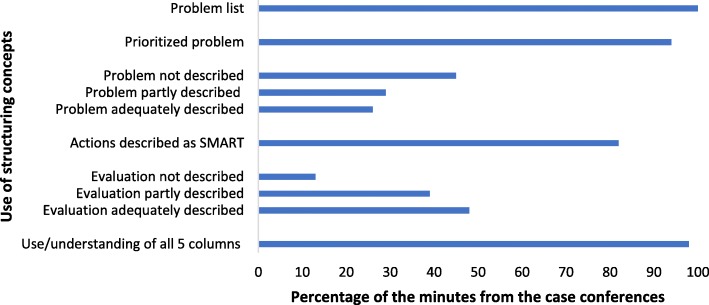
Documentary analysis of minutes from 84 case conferences. Notes: SMART, Specific, Measurable, Actual, Realistic, Time framed

The results from the documentary content analyses of the 84 min of the case conferences are illustrated in Fig. 1. The description of the problems analysed during the case conferences (i.e. description in detail of symptoms or behaviour) was performed adequately in 22 (26%) of the case conferences. For the description of the evaluation procedures this was done adequately in 49 (48%) of the case conferences. For the other structuring components of the case conferences, adequately understanding and use of them varied among the components from 69 (82%) to 84 (100%) of the minutes.

### Maintenance - facilitators and inhibitors to the implementation process

All but one of the 11 nursing homes represented in the five focus groups were still using the model 3 to 6 months after the end of the intervention. The frequency of the case conferences varied from once a week to once a month. The nursing home that had stopped to use the model, did so because they had moved all their residents to a new building. They considered re-implementing TIME as soon as they settled in. Several factors were considered when the focus groups discussed what promoted and what inhibited the maintenance of the model. These findings are summarised in Table 7.

**Table 7:** Findings from analyses of focus groups: Excerpt from the development of the theme: facilitators and barriers for maintenance

Sub-themes	Codes	Condensed meaning units	Meaning units
An easy to grasp model	Simple to understand Immediate result of their efforts for the residents	A Simple and manual based model A tool that works	“It is all written, black on white, step by step, and so you have these forms, so it is easy to use, yes, it is a simple system” (Staff). “Then you have these columns where you write things, and it makes it easier….to understand the whole situation for the resident” (Staff). “And we saw that those residents included in the study, did get a better everyday life, we put more emphasis on what is important for them. So, yes, we can see that we have changed the way we care for them” (TIME-administrator).
The role of leadership	Leader	The leader is responsible in prioritising time and space for the assessments and the case conferences, and to follow up and ensure that the tasks are executed	(4). “I had to push them a bit. Well, they did know about the TIME model, or the schedule, and everything they were supposed to do, but as a leader you must facilitate or else they won’t get it done. Yes, I think I must allow them the space. Tell them that now you should spend time on this” (Leader)
	The TIME-administrators (champions) role as leaders	The importance of being an engaged team of TIME-administrators (champions).	(3). “At my ward, we are four TIME-administrators. So, in a way, we are many to pull the load. So, I think we do well, because we are engaged. It is not just *one* person that has to work hard” (TIME-administrator)
	Training is given to everyone	The importance that the leader included everyone in the educational program of the model	(4). “I think it is crucial that everybody learned the same thing, that everyone participated. And it was also a bit developing our own competence. In that way, I included temps... so, it is much easier, when everyone has heard about it” (Leader)
	Organisational conditions	Participation in the case conference should be compensated	(4). “The TIME model is so important, that if we are going to succeed, we must give them time off. For we cannot expect them to attend the case conference in their spare time” (Leader)

One important condition for using the model was that it was considered simple to learn, to understand, and to start using. Another condition was that they could see an immediate result of their efforts for the residents.

All the groups believed that the intervention had to be properly supported by the leading registered nurse. Time allocated and active support by the leader for assessment tasks was important. In addition, the staff thought that the leaders ought to actively give support in the case conferences and support for follow-up and evaluation procedures. It was deemed vital that as many staff members as possible could participate in the initial educational program. This was important for creating acceptance among all the staff for this way of working. The TIME administrator’s role was highlighted by both the staff and the leaders, and by themselves as essential for the implementation process. At the same time as they were an integrated part of the staff, they also felt a commitment to engage each other as a group, and the rest of the staff in the work to implement the different components of the model.

A theme that was recurring was the difficulty encountered in arranging and finding time for the case conferences. These difficulties were regarded as the main barrier to a proper implementation of the model. Here too, wards with an engaged leader who saw the benefits of the intervention, did better. The leaders in these wards were able to incorporate the case conferences as a part of the routines in the wards. The participants also reported better attendance at the case conferences in these wards. In other nursing homes, attending the case conference was voluntary for the staff who were not on duty. Some nursing homes did not provide compensation for the attendance on free time. Several participants attributed varying attendance at meetings and varying frequency of the case conferences to these factors and to the leader’s engagement.

## Discussion

Our results revealed a high degree of reach, adoption, and implementation of the intervention with TIME. Effectiveness at staff level measured by competence (i.e. general knowledge on NPS and skills), general attitudes towards dementia, mastery and social interaction at the working place, did not differ between the INH and the CNH. The analyses of the qualitative data showed that an easy to grasp model and a present and an engaged leadership are important facilitators for maintenance. We will discuss these results in the light of the RE-AIM dimensions and analyse to which extent these results can contribute to assumptions of causal mechanisms of the effectiveness of TIME at resident level**.**

### Reach, adoption and implementation

The importance of aiming to train most staff (i.e. high degree of reach) was highlighted by Rapaport et al. (2017) in a systematic review on the effective components of psychosocial interventions [64]. In this review, staff perceived difficulties sharing new approaches with those who had not attended training sessions.

A high degree of adoption and implementation in our study can to some extent be explained by the finding that TIME was perceived by the staff as an easy to grasp model. To achieve acceptable adoption and implementation, several studies support the importance of interventions to be simple, appreciated by the staff, and not too overly complex [37–39].

Though implementation and adoption were high in our study, TIME was used with some variation between the study sites. The variation in the time the staff needed to perform the assessment of each resident, how long it took them to organise a case conference, and how many of the staff from each ward that attended case conferences, may in part be explained by organizational conditions. Allowing for some adaption of complex interventions to the organisational context of the settings is in line with viewing organisations (e.g. nursing homes) as complex systems. This flexibility of a complex intervention has been emphasized as an important factor to improve the effectiveness of the interventions by increasing their applicability [16, 35, 42].

The INH and the CNH did not differ throughout the study period regarding adherence to recommended clinical practice towards NPS as measured by the Current Practice Questionnaire (see Table 5). This can be due to several reasons. Firstly, participants in a research project could be affected by the so-called Hawthorne effect meaning that the participants change their behaviours only because they are monitored in a study [65]. Secondly, the questionnaire is based on self-reporting of practices by the leading ward nurses. Self-reporting of behaviour change in these settings is prone to bias [17]. It is possible that leaders from both groups gave more favourable answers than what are reflected in their practices. This could have offset any differences between the two groups. Thirdly, both groups had similar high scores at baseline, indicating that they already followed most of these recommendations providing less possibilities for improvement. Finally, both groups received lectures on NPS and recommended clinical practice for the approach towards NPS. Since both groups significantly increased their scores on this questionnaire throughout the study, this could indicate that both groups followed, to some degree, the recommendations from these lectures. As will be further discussed in the next paragraph, the effectiveness of TIME at resident level might to a lesser degree depend on the clinical practice routines.

### Effectiveness

Though there was an increased level of positive attitudes towards dementia in both groups, and an increased level of knowledge in the intervention group, there were no between-group differences in any measurements of effectiveness at individual staff level throughout the study period. These results are in line with the results in two recent systematic reviews on the impact of staff training on staff outcomes [40, 43]. However, in the same reviews some studies found increased staff knowledge and self-efficacy, but these findings were not consistently maintained over time. On the other hand, our recently published report exploring in depth the staff’s learning and coping experiences with TIME, showed that the staff perceived an increase in specific knowledge and coping related to each individual resident [32] . These changes were interpreted as a consequence of a new innovative learning process that took place during the interdisciplinary case conferences. This learning process created a new and shared situated knowledge about the resident and made it easier to customise the approach towards the individual resident. Most questionnaires assess mainly general attitudes and general knowledge about dementia [66]. These general attitudes and knowledge do not necessarily reflect the staff’s capability to translate this general competence into their real-world approach to an individual resident with, for example, severe agitation by which they are confronted on an everyday basis. The creation of a new, shared and situated knowledge about each resident might be one of the most important contributions to the effectiveness of TIME at staff level.

### Maintenance - facilitators and inhibitors to the implementation process

Our data suggest that most of the nursing homes were still using TIME 3 to 6 months after the end of the intervention. Adherence to recommended clinical practice was sustained after 12 months. In accordance with our results from the focus groups, process evaluations of complex interventions in nursing homes highlights the importance of management support to achieve both adoption and sustainability [37–40, 43, 64, 67]. In the systematic review from Rapaport et al. (2017), a leadership attending training sessions and meetings, and allocating space and time for the staff to engage in the intervention, was conceived as one of the most important facilitators for a sustainable implementation [64]. Most of the leading registered nurses in the TIME trial attended the same educational training sessions as the rest of the staff and they also attended nearly half of the case conferences. The finding that the TIME administrators were important for the implementation process and acted as implementation leaders, is in line with other studies [68–71]. Scalzi et al. (2006) used the label “change champions” and regarded them one of the most important enablers for culture change [69].

As discussed for adoption and implementation, the staff expressed that TIME was an easy to grasp model. They emphasised that this feature had an impact on the possibility for continuing using the model. In the trial, after a short education and training programme, the TIME administrators in cooperation with the leading ward nurse carried out the intervention independently of the research team. This will increase the probability for maintenance of the intervention, reduce the cost for implementation and ease the proliferation of the model.

Turnover of care staff is often reported as a main barrier to sustainable interventions in nursing homes [64, 72]. However, turnover did not emerge as a subtheme in the focus group discussions when raising questions about barriers to the implementation process. The high degree of attendance to training sessions from all staff including those working part-time and with temporary positions, participation of the leading ward nurses in the training sessions, selecting three TIME-administrators among the nursing home staff as implementation champions and an easy-to-grasp model may have outweighed some of the effect of staff turnover.

### Limitations and strengths

The main strength of this process evaluation is the rigorous use of a well-established and recommended framework for evaluation of complex interventions [42, 44]. The RE-AIM framework mainly emphasises quantification of the five dimensions reach, effectiveness, adoption, implementation, and maintenance to explore what is called the public health impact of the process of implementation [42, 44]. Less emphasis is put on the process of implementation. We have therefore in our study included issues regarding facilitators and barriers of the implementation process and considerations on causal assumptions of the effectiveness of the intervention [42].

The only interference during the randomised controlled trial was the use of the checklist for assessing performance of the main components of the intervention to explore implementation (fidelity). This checklist could have possibly served as a reminder for the TIME administrators.

During this process evaluation we have used a quasi-experimental design giving us the same quantitative data from the control condition as from the intervention group. Another strength is that we used a mixed-method design which allows us to compare and interpret quantitative results from the survey with findings from the perspectives of the staff, leaders, and physicians derived from the focus groups. This comparison adds to the validity of our results and to the value of our assumptions on possible causal mechanisms of the effect of TIME at resident level [41, 46].

One important limitation is that we do not know to which extent the model became a stable enduring part of the clinical routines of the nursing homes beyond 6 months after the end of the intervention.

One other limitation is that this process evaluation was performed by the same research team as the team responsible for the trial [42]. It was also performed when results from the trial were known, even though all the data collection for the process evaluation were planned for in a published protocol, and collected independently of the trial [18]. Both these phenomena could have created some bias in the analysis of the data. On the other hand, integrating process and outcomes’ evaluation by the same team, may limit the risk of one collection of data disturbing the other.

The relatively low response to some of the questionnaires is also an important limitation. One of the reasons for this is probably that the nursing home staff did not regularly use the e-mail account at work. We do not have data on the part of the staff who did not participate in the survey. This limits the external validity of the results. However, a recently published study comparing staff characteristics in special care units and regular units in Norway did find similar staff characteristics as in our sample [73].

The questionnaires to the staff members at 6 months and at 12 months were only administered to those of the staff wo had answered the questionnaires at baseline and at 6 months, respectively. Given the staff turnover within nursing homes, this procedure of data collection limits our possibility to draw conclusions on the impact of the intervention on the whole group of staff members throughout the trial and on an eventually pass on effect of the intervention among the staff members. Future process evaluations of interventions in care settings, should explore also this aspect of an intervention since this might be crucial for the intervention’s sustainability.

The successive reduction of responders at 6 months and at 12 months may also have influenced the results on the measured outcomes. It is possible that those who continued to respond are more positive to the intervention and in general more interested in professional development. However, the response rates were similar in both INH and CNH throughout the study period. Assuming that the characteristics of the staff that did not participate in the survey and their reasons for not participating are equally distributed in the two groups, the comparison between the two groups should, however, be judged as valid.

Two of the of questionnaires in the study were developed by the research team to be brief and to evaluate the implementation process inherent to TIME. With these questionnaires we wanted to evaluate the self-reported competence of the staff members and the clinical routines in the NH regarding the approach especially towards NPS, not towards dementia in general. Because of these requirements we did not find other equivalent questionnaires. They were not validated, and their psychometric properties are not known. This is an important limitation in this process evaluation study. However, since the questionnaires are carefully developed to reflect the content of the lectures and training sessions in both the INH and the CNH, we do believe they measure, at least to some extent, what the staff members eventually have learned from these lectures and training sessions in both the INH and the CNH. This is supported by the finding that perceived competence of the staff members increased significantly from baseline to 12 months in the INH, but not in the CNH. The Fidelity Questionnaire is a check-list for the performance of the main components in TIME, conducted by interviewing the TIME administrators by telephone, and is, therefore, less prone to self-reporting bias. There is still a concern about the accuracy of self-reporting of behaviours without checking with objective fidelity measures [17]. However, the documentary analysis of the meetings from the case conferences in our study contributed to a more valid judgement of the implementation (fidelity) dimension of the RE-AIM framework.

## Conclusion

A high degree of reach, adoption, implementation, and maintenance have probably contributed to the effectiveness of TIME at resident level. Effectiveness measured by changes in general attitudes and competence at an individual staff level, and in clinical routines at ward level, did not reveal differences between the intervention nursing homes and the control nursing homes. An easy to grasp model and an engaged and a present leadership were the most important facilitators for maintenance. Lack of support from the leading ward nurse, and not integrating the case conferences as a part of the routines in the wards, were perceived as the main barriers to implementation and maintenance. Another causal assumption of the effect observed at resident level, is that TIME contributed to the development of a new, shared and situated knowledge in the staff about each individual resident and the actual NPS. Future process evaluations on complex interventions should explore methods for assessing how the staff’s general knowledge and attitudes are translated into their every-day approach to each individual resident.

## Additional files

Additional file 1: Questionnaires to the staff. (DOCX 30 kb)

Additional file 2: Methods used for qualitative data collection and analysis. (DOCX 35 kb)

## Data Availability

The quantitative data supporting the conclusions of this article are available on reasonable request to the first author. The transcribed interviews are due to confidentiality, not available.

## Abbreviations

ADQ: Approaches to Dementia Questionnaire
BPSD: behavioural and psychological symptoms in dementia
CBT: cognitive behavioural therapy
CDR: Clinical Dementia Rating Scale
CI: Confidence Interval
CMAI: Cohen-Mansfield Agitation Inventory
CNH: Control nursing homes
CSDD: Cornell Scale of Depression in Dementia
GMHR: General Medical Health Rating Scale
INH: Intervention nursing homes
MMSE: Mini-Mental State Examination
MOBID-2: Mobilisation-Observation-Behaviour-Intensity-Dementia Scale
MRC: Medical Research Council
NPI-NH: Neuropsychiatric Inventory-Nursing Home Version
NPS: Neuropsychiatric symptoms
PSMS: Physical Self-Maintenance Scale
QPS: General Nordic Questionnaire for Psychological and Social Factors at Work
RE-AIM: Reach-Effectiveness-Adoption-Implementation-Maintenance
SD: Standard deviation
SD: Standard deviation
SMART: Specific-Measurable-Actual-Realistic-Time
SPSS: Statistical Product and Service Solution
TIME: Targeted Interdisciplinary Model for Evaluation and Treatment of Neuropsychiatric Symptoms
