# Supplementary material for: TIME to reduce agitation in persons with dementia in nursing homes. A process evaluation of a complex intervention
Source: BMC Health Serv Res. 2019 May 31;19:349. doi: 10.1186/s12913-019-4168-0 (PMC6544967; doi:10.1186/s12913-019-4168-0)
Supplement: Supplementary file 1 — Questionnaires to the staff. (DOCX 30 kb) [file 12913_2019_4168_MOESM1_ESM.docx]

**Additional file 1**

**Questionnaires to the staff**

**TIME^1^ to reduce agitation in dementia in persons with dementia in nursing homes. A process evaluation of a complex intervention**

^1^TIME: Targeted Intervention Model for Evaluation and Treatment of Neuropsychiatric Symptoms

**Approach to Dementia Questionnaire (ADQ) [1, 2]**

This questionnaire was distributed to the staff one month before, six and twelve months after the start of the intervention in both the intervention nursing homes and control nursing homes. The total Range for ADQ is 19-95, a higher score indicating better attitudes towards dementia. Factor analysis has shown that the items 1-4, 6, 8, 10, 13 correspond to the hope attitude dimension (Range 8-40) and items 5, 7, 9, 11, 12, 14-19 correspond to the person-centred caring attitude dimension (Range 11-55) [2]. Since the Likert scale in the questionnaire is inversed for some of the items in the two dimensions, the entire questionnaire with the Likert scale is presented below.[

**Introduction to the questionnaire for the respondents:**

Below you will find some statements that explore your general attitudes towards persons with dementia. Make a mark by the answer that suits you the best. For each statement there will be persons who will agree or disagree, so there are no right or wrong answers. Try to answer as quickly as possible, the first alternative that feels naturally, is often the one that suits you the best.

|  | **Approach to Dementia Questionnaire (ADQ)** | Strongly agree | Agree | Neither agree or disagree | Disagree | Strongly  disagree |
| --- | --- | --- | --- | --- | --- | --- |
| 1 | It is important to have a very strict routine when working with dementia sufferers | 1 | 2 | 3 | 4 | 5 |
| 2 | People with dementia are very much like children | 1 | 2 | 3 | 4 | 5 |
| 3 | There is no hope for people with dementia | 1 | 2 | 3 | 4 | 5 |
| 4 | People with dementia are unable to make decisions for themselves | 1 | 2 | 3 | 4 | 5 |
| 5 | **It is important for people with dementia to have stimulating and enjoyable activities to occupy their time** | 5 | 4 | 3 | 2 | 1 |
| 6 | Dementia sufferers are sick and need to be looked after | 1 | 2 | 3 | 4 | 5 |
| 7 | **It is important for people with dementia to be given as much choice as possible in their daily lives** | 5 | 4 | 3 | 2 | 1 |
| 8 | Nothing can be done for people with dementia, except for keeping them clean and comfortable | 1 | 2 | 3 | 4 | 5 |
| 9 | **People with dementia are more likely to be contented when treated with understanding and reassurance** | 5 | 4 | 3 | 2 | 1 |
| 10 | Once dementia develops in a person, it is inevitable that they will go down hill | 1 | 2 | 3 | 4 | 5 |
| 11 | **People with dementia need to feel respected, just like anybody else** | 5 | 4 | 3 | 2 | 1 |
| 12 | **Good dementia care involves caring for a person’s psychological needs as well as their physical needs** | 5 | 4 | 3 | 2 | 1 |
| 13 | It is important not to become too attached to residents | 1 | 2 | 3 | 4 | 5 |
| 14 | It doesn’t matter what you say to people with dementia because they forget anyway | 1 | 2 | 3 | 4 | 5 |
| 15 | **People with dementia often have good reasons for behaving as they do** | 5 | 4 | 3 | 2 | 2 |
| 16 | **Spending time with people with dementia can be very enjoyable** | 5 | 4 | 3 | 2 | 1 |
| 17 | **It is important to respond people with dementia with empathy and understanding** | 5 | 4 | 3 | 2 | 1 |
| 18 | **There are a lot of things that people with dementia can do** | 5 | 4 | 3 | 2 | 1 |
| 19 | **People with dementia are just ordinary people who need special understanding to fulfil their needs** | 5 | 4 | 3 | 2 | 1 |

**General Questionnaire for Psychological and Social Factors at Work (QPS-Nordic) (3]**

This questionnaire was distributed to the staff 1 month before, 6 and 12 months after the start of the intervention in both the Intervention Nursing Homes and Control Nursing Homes. The answers are given on a 5-point Likert scale; Very seldom or never:1; Rather seldom:2; Sometimes:3; Rather often:4; Very often or always:5. This will give a sum range, 6-30, for each of the two sub scores, a higher score indicates better mastery at work or better social interactions and support.

**Introduction to the questionnaire for the respondents:**

No specific instructions were given to the respondents.

|  | Mastery at work |
| --- | --- |
| 1 | Are you content with the quality of the work you do? |
| 2 | Are you content with the amount of work that you get done? |
| 3 | Are you content with your ability to solve problems at work? |
| 4 | Are you content with your ability to maintain a good relationship with your co-workers at work? |
| 5 | Do you get information about the quality of the work you do? |
| 6 | Can you yourself immediately assess whether you did your work well? |
|  | **Social interaction** |
| 1 | If needed, can you get support and help with your work from your co-workers? |
| 2 | If needed, can you get support and help with your work from your immediate superior? |
| 3 | If needed, are your co-workers willing to listen to your work-related problems? |
| 4 | If needed, is your immediate superior willing to listen to your work-related problems? |
| 5 | Are your work achievements appreciated by your immediate superior? |
| 6 | Have you noticed any disturbing conflicts between co-workers? |

**Self-assessment of competence about neuropsychiatric symptoms (NPS).**

This is a self-developed questionnaire assessing individual competence about NPS. It was distributed to the staff 1 month before, 6 and 12 months after the start of the intervention in both the intervention nursing homes (INH) and control nursing homes (CNH) Competence was defined as the combination of knowledge and skills [4]. The answers are given on a 7-point Likert scale: Very low:1; Low:2; Quite low:3; Neither low or high:4; Quite high:5; High:6; Very high:7. This will give a sum range, 5-35, a higher score indicates better competence.

**Introduction to the questionnaire for the respondents:**

We will now ask you to make your own judgements about your competence in your work with persons with dementia who present challenging behaviour. With competence in this regard, we mean the sum of your knowledge and skills as judged by you.

| 1 | I consider my competence in contributing to the assessment of patients with dementia and challenging behaviour as: |
| --- | --- |
| 2 | I consider my competence in interpreting challenging behaviour in patients with dementia as: |
| 3 | I consider my competence in contributing to treatment measures for patients with dementia and challenging behaviour as: |
| 4 | I consider my competence in evaluating treatment measures for patients with dementia and challenging behaviour as: |
| 5 | I consider my competence in the approach to patients with dementia and challenging behaviour as: |

**The Current Practice Questionnaire**

This questionnaire assesses daily routines of practice for assessment and treatment of neuropsychiatric symptoms (NPS) at ward level. This is a self-developed questionnaire distributed to the leading ward nurse in the intervention nursing homes and the control nursing homes, 1 month before and 6 and 12 months after the start of the intervention. It is based on evidence-informed best practice for the assessment and the treatment of NPS [5]. The scale used is a 5-point Likert scale: Very seldom or never:1; Rather seldom:2; Sometimes:3; Rather often:4; Very often or always:5. This gives a total sum range from 13-65, a higher score reflecting better adherence to recommended practice for the assessment and treatment of neuropsychiatric symptoms.

**Introduction to the questionnaire for the respondents:**

All the questions concern the routines followed by the ward or unit on a daily basis, i.e. what you perform in your daily practice when you have residents with dementia presenting challenging behaviour.

| 1 | To assess or follow severe Behavioural and Psychological Symptoms (BPSD), how often do you use a 24-hour observation form? |
| --- | --- |
| 2 | For residents with severe BPSD, how often do you assess their level of functioning in daily life activities? |
| 3 | How often do you use the Neuropsychiatric Inventory (NPI) to assess symptoms and behaviour for residents with severe BPSD? |
| 4 | How often are residents with severe BPSD examined by a physician as a part of the assessment? |
| 5 | How often do you assess the residents’ personal life history, including habits and preferences, as a part of the assessment of severe BPSD? |
| 6 | How often do you perform a systematic review of the residents’ medication when they have severe BPSD? |
| 7 | For residents with severe BPSD, how often do you use an assessment form or scale to assess if the patient might have a depression (Cornell Scale of Depression in Dementia or another scale? |
| 8 | For residents with severe BPSD, how often do you perform a systematic assessment of pain, for example by the physician or by the staff using a pain assessment form (for example MOBID-2) |
| 9 | How often do you perform an assessment for possible dementia for residents with severe BPSD who have not yet been diagnosed? |
| 10 | For residents with severe BPSD, how often do the staff alone or in cooperation with the physician, perform an assessment of the degree of dementia by using assessments scales like MSSE, Clinical Dementia Rating Scale (CDR) or other equivalent scales? |
| 11 | For residents with severe BPSD, how often do the staff gather in meetings, case conferences etc. to discuss the assessment and to arrive at joint actions and measures? |
| 12 | When the staff have set up a treatment plan for residents with severe BPSD, how often is this plan put into action and followed as planned? |
| 13 | How often do you at meetings perform an evaluation of the measures and treatment plans you have put into action for residents with severe BPSD? |

**The Fidelity Questionnaire**

|  | Questions | Score | |
| --- | --- | --- | --- |
| 1 | Are you using the 24-hour observation form for the resident? | yes=1 | no=0 |
| 2 | Have you assessed the resident using the Neuropsychiatric Inventory? | yes=1 | no=0 |
| 3 | Have you obtained the personal life story of the resident? | yes=1 | no=0 |
| 4 | Have you assessed depressive symptoms with the CORNELL Scale or another scale for the assessment of depression? | yes=1 | no=0 |
| 5 | Have you assessed the resident’s level of functioning in daily life activities with PSMS or another equivalent scale? | yes=1 | no=0 |
| 6 | Have the resident been assessed for pain? | yes=1 | no=0 |
| 7 | Have you assessed the level of dementia with the Clinical Dementia Rating scale or any equivalent scale? | yes=1 | no=0 |
| 8 | Has a physician examined the resident? | yes=1 | no=0 |
| 9 | Has there been conducted a case conference about the resident? | yes=8 | no=0 |
| 10 | Have you performed a systematic evaluation of the measures decided upon in the case conferences? | Yes=4 | no=0 |

This questionnaire is based on the check-list of the main components of TIME as they are described in the TIME manual [6]. The TIME administrators were interviewed briefly for each resident included in the trial. This was done by the research team by telephone three times during the 12 weeks trial, with three to four weeks interval. The different components have been given a weighted score based on the presumed time spend to perform each component. The sum score ranges from 0-20, a higher score signifies better fidelity (implementation) to the model. In the manuscript we have reported the percentage performed of the main components for the residents based on this score, where the maximum score of 20 signifies that 100 percent of the components were performed for a resident.

**References**

1. Kada S, Nygaard HA, Mukesh BN, Geitung JT. Staff attitudes towards institutionalised dementia residents. J Clin Nurs. 2009;18(16):2383-92.

2. Lintern T. Quality in Dementia Care: Evaluating Staff Attitudes and Behaviour.: University of Wales Bangor; 2001.

3. Dallner M, Elo A-L, Gamberale F. Validation of the General Nordic Questionnaire (QPSNordic) for psychological and social factors at work. Copenhagen: Nordic Council of Ministers; 2000.

4. Bing-Jonsson PC, Bjork IT, Hofoss D, Kirkevold M, Foss C. Competence in advanced older people nursing: development of 'nursing older people--competence evaluation tool'. Int J Older People Nurs. 2015;10(1):59-72.

5. Kales HC, Gitlin LN, Lyketsos CG. Management of neuropsychiatric symptoms of dementia in clinical settings: recommendations from a multidisciplinary expert panel. J Am Geriatr Soc. 2014;62(4):762-9.

6. Lichtwarck B, Tverå AM, Røen I. TIME - Targeted Interdiciplinary Model for Evaluation and treatment of neuropscychiatric symptoms - Manual 2nd Edition. Ottestad (Norway): The Research Centre for Age-related Functional Decline and Disease - Innlandet Hospital Trust; 2015 [30.04.2017]. Available from: [www.tidmodell.no](http://www.tidmodell.no). Accessed 30 Apr 2017.
