# Supplementary material for: TIME to reduce agitation in persons with dementia in nursing homes. A process evaluation of a complex intervention
Source: BMC Health Serv Res. 2019 May 31;19:349. doi: 10.1186/s12913-019-4168-0 (PMC6544967; doi:10.1186/s12913-019-4168-0)
Supplement: Supplementary file 2 — Methods used for qualitative data collection and analysis. (DOCX 35 kb) [file 12913_2019_4168_MOESM2_ESM.docx]

**Additional file 2**

**Methods used for qualitative data collection and analysis**

**TIME^1^ to reduce agitation in persons with dementia in nursing homes. A process evaluation of a complex intervention**

^1^TIME: Targeted Intervention Model for Evaluation and Treatment of Neuropsychiatric Symptoms

*Data collection*

*Focus groups*

Three to six months after the end of the intervention, five focus groups interviews with 32 of the caregivers, leaders, and physicians from 11 of the 17 intervention nursing homes (INH) were conducted [1]. The nursing homes that were represented in the groups were selected randomly from the pool of INH in order to minimize selection bias [2]. The composition of the groups was as follows: two staff groups with eighth and six informants consisting of staff members, registered nurses and auxiliary nurses (where two staff members together in each group came from the same nursing home); one TIME administrator group with eight registered nurses and auxiliary nurses; one leader group with seven registered leading ward nurses and one physician group with three nursing home physicians (one participant from each nursing home in these last three groups).

To achieve information-rich cases and a purposeful sample, the leading ward registered nurse in each nursing home selected who of the staff would attend the focus groups [3]. The criteria for the selection of participants were that they should be familiar with TIME and be able to promote views in a group discussion. The interviews were all recorded and transcribed verbatim by the first and second author.

According to the study protocol of the TIME trial, we also used these focus groups interviews to collect data to explore the staff’s learning and coping experiences with TIME and how the model meets the challenges when dealing with the complexity of neuropsychiatric symptoms (NPS) [4]. This is also reflected in the interview guide, see table 2 in this Appendix. These results are published in a separate paper (5] . Background characteristics of the participants and the wards in the focus groups are presented in Table 2 in this Appendix.

Each group met once for a 90-minute interview in a meeting room in a hotel. The moderator was BL for three of the interviews and JM for two of the interviews. They were both present in all five interviews as a moderator or a facilitator. A third researcher, not affiliated with the research team for this project, participated in three interviews, and served as a co-facilitator. She posed follow-up questions towards the end of each interview. The interviews were based on a semi-structured interview guide where the informants were asked to reflect on two main themes [6]: 1) Coping and learning in working with residents with dementia and NPS and 2) Implementation and sustainability of the TIME intervention. These main themes were discussed by using open-ended and exploratory questions. Other themes that emerged spontaneously were also discussed. At the end of each interview, the facilitator summarised the explicit content of the interview and the participants were asked to verify or correct the summary.

*Minutes from case conferences*

To further assess implementation we collected the minutes from 84 of the 85 case conferences in the INH [7, 8]. The minutes were all written by one staff member from each ward during the case conference using the 5-column sheet for problem analysis from the TIME manual [9]. The use of the 5-column sheet for problem analysis were part of the training sessions for the staff in the intervention nursing homes. The main purpose of writing minutes during the case conferences, was to create a written documentation to be integrated in the residents’ care plans.

*Data analysis*

*Focus groups*

For the analysis of the data for the focus groups, thematic content analysis was used. In thematic content analysis the purpose is to identify, analyse, and reveal themes in qualitative data [10, 11]. Both the manifest and latent content of the data are systematically described, and new concepts and understanding of phenomena are developed. This analysis was performed in four steps: (1) an overview of the content was obtained from multiple readings of the transcribed text; (2) meaning units were identified using coding, and these meaning units were then condensed. Coding was done by labelling related text elements, derived from the original text, and then meaning units were reassembled in a new document; (3) these units were then abstracted and grouped into subthemes and the subthemes that were related; and (4) these subthemes and themes were discussed in the context of our research questions, existing theory or new theoretical formulations, if necessary [10]. This approach to the coding process was mainly inductive, and we therefore coded the entire data set [10].

The primary analysis was performed by BL and JM, while the third researcher who participated in the interviews, did the primary analysis alone before discussing her results with the rest of the authors. Preliminary analyses were conducted after each group, and when no obvious new meanings units or new sub-themes occurred in the last interview, we considered having met saturation of the data [12].

*Minutes from case conferences*

The documentary analysis of the minutes from 84 case conferences were performed by using structuring documentary content analysis [7, 8]. This analysis looks for types or formal structures in the data and uses preformed categories as codes to analyse frequencies and different degrees of quality in a category. This approach to the coding process is deductive, and therefore does not analyse the documents for other content, patterns, or themes. We will in subsequent paper report from the results of a documentary analysis using a more inductive approach with thematic analysis of the minutes. According to our research question which concerns the fidelity to the model, our categories were the main structuring components of the case conferences used in TIME [9]. For the components: use of a problem list, selection of a prioritized problem, the overall understanding and use of the five columns, and actions described as SMART, these were classified as being performed or not. For the descriptions of the components: prioritized problem and evaluation procedures of treatment actions, these were classified by the degree of details in the description (adequately, partly, or not described). The first and second authors separately evaluated each minute according to this procedure and met to achieve a consensus. A frequency analyses of the minutes were then performed based on these categories. The results from these analyses are presented in the paper in figure 2.

**References**

1. Krueger RA, Casey MA. Focus groups: A practical guide for applied research. Los Angeles: Sage publications; 2015.

2. Krueger RA, Casey MA. Answering questions about the quality of focus goups research. In: Krueger RA, Casey MA, editors. Focus groups: A practical guide for applied reserach. Los Angeles: Sage publications; 2015. p. 237-45.

3. Patton M. Designing qualitative studies. In: Patton M, editor. Qualitative research and evaluation methods. United States of America: Sage Publications, Inc.; 2015.

4. Lichtwarck B, Selbaek G, Kirkevold O, Rokstad AM, Benth JS, Myhre J, et al. TIME - Targeted interdisciplinary model for evaluation and treatment of neuropsychiatric symptoms: protocol for an effectiveness-implementation cluster randomized hybrid trial. BMC Psychiatry. 2016;16(233):1-12.

5. Lichtwarck B, Myhre J, Goyal AR, Rokstad AMM, Selbaek G, Kirkevold O, et al. Experiences of nursing home staff using the targeted interdisciplinary model for evaluation and treatment of neuropsychiatric symptoms (TIME) - a qualitative study. Aging Ment Health. 2018:1-10.

6. Kvale S, Brinkmann S. Interviews: Learning the craft of qualitative research. Los Angeles: Sage Publications; 2009.

7. Bowen GA. Document analysis as a qualitative research method. Qualitative research journal. 2009;9(2):27-40.

8. Flick U. Thematic Coding and Content Analysis. In: Flick U, editor. An introduction to qualitative research. London: SAGE Publications Inc.; 2014. p. 420-38.

9. Lichtwarck B, Tverå AM, Røen I. TIME - Targeted Interdiciplinary Model for Evaluation and treatment of neuropscychiatric symptoms - Manual 2nd Edition. Ottestad (Norway): The Research Centre for Age-related Functional Decline and Disease - Innlandet Hospital Trust; 2015 [30.04.2017]. Available from: [www.tidmodell.no](http://www.tidmodell.no). Accessed 30 Apr 2017.

10. Braun V, Clarke V. Using thematic analysis in psychology. Qualitative research in psychology. 2006;3(2):77-101.

11. Vaismoradi M, Turunen H, Bondas T. Content analysis and thematic analysis: Implications for conducting a qualitative descriptive study. Nurs Health Sci. 2013;15(3):398-405.

12. Malterud K. Qualitative research: standards, challenges, and guidelines. Lancet. 2001;358(9280):483-8.

**Table 1 (Appendix 2). Background characteristics of the participants (n=32) and the wards (n=12) in the focus groups.** Values are numbers (%), unless otherwise specified. SD; Standard deviation. Participants came from 11 nursing homes with one nursing home represented with two wards.

| Background characteristics | Number (%) |
| --- | --- |
| **Age (years)** |  |
| ≤29 | 5 (16) |
| 30–49 | 14 (44) |
| ≥ 50 | 13 (40) |
| **Sex** |  |
| Female | 29 (91) |
| **Working experience in years in health-related job** |  |
| <1 | 0 (0) |
| 1–5 | 5 (16) |
| 6–10 | 6 (19) |
| 11–15 | 1 (3) |
| >15 | 20 (62) |
| **Profession** |  |
| Auxiliary nurse | 12 (38) |
| Registered nurse | 10 (31) |
| Leading ward registered nurse | 7 (22) |
| Physician  **Wards**  Regular ward  Special care unit  Residents per ward, Mean (SD)  Staff per resident on day shift, Mean (SD)  Hours per resident per week for nursing home physician, Mean (SD) | 3 (9)  2 (17)  10 (83)  21.8 (9.6)  0.39 (0.13)  0.36 (0.15) |

**Table 2 (Appendix 2) The interview guide.** Results from theme 2 are presented in this paper. Results form theme 1 was published in in a separate paper [5].

| Themes | Questions used in the interviews |
| --- | --- |
| 1. Coping and learning in working with residents with dementia and NPS | What are your thoughts/views about your own knowledge in your work with residents with dementia and neuropsychiatric symptoms?  Same question as above posed for attitudes, skills, and coping. |
|  | Same questions as above, but now their thoughts/views concerning the rest of the staff. |
|  |  |
|  | Has using TIME affected your own knowledge in your work with residents with dementia and neuropsychiatric symptoms? If so, in what way?  Same question posed for attitudes, skills, and coping. |
|  | Same questions as above: but now their thoughts/views concerning the rest of the staff. |
|  |  |
| 1. Implementation and sustainability of the intervention | Which conditions are of significance to adopt a model like TIME in your ward?  What promotes and what inhibits the adoption of such a model? |
|  | Conditions in the ward? |
|  | Conditions concerning the education and training program for the model? |
|  | Conditions concerning the model itself? |
|  |  |
|  | Which conditions are of significance to continue to use a model like TIME in your ward? |
|  | Conditions in the ward? |
|  | Conditions concerning the education and training program for the model? |
|  | Conditions concerning the model itself |
